# Cortical thickness and surface area as an endophenotype in bipolar disorder type I patients and their first-degree relatives

**DOI:** 10.1016/j.nicl.2019.101695

**Published:** 2019-01-29

**Authors:** Nefize Yalin, Aybala Saricicek, Ceren Hidiroglu, Andre Zugman, Nese Direk, Emel Ada, Berrin Cavusoglu, Ayşe Er, Gizem Isik, Deniz Ceylan, Zeliha Tunca, Matthew J. Kempton, Aysegul Ozerdem

**Affiliations:** a Centre for Affective Disorders, Department of Psychological Medicine, Institute of Psychiatry, Psychology & Neuroscience, King's College London, London, UK; b Department of Neuroscience, Institute of Health Sciences, Dokuz Eylul University, Izmir, Turkey; c Department of Psychiatry, Faculty of Medicine, Katip Celebi University, Izmir, Turkey; d Department of Psychology, Faculty of Arts, Dokuz Eylul University, Izmir, Turkey; e Interdisciplinary Laboratory of Clinical Neuroscience (LINC), Department of Psychiatry, Universidade Federal de Sao Paulo, Sao Paulo, Brazil; f Department of Radiology, Faculty of Medicine, Dokuz Eylul University, Izmir, Turkey; g Department of Psychiatry, Faculty of Medicine, Dokuz Eylul University, Izmir, Turkey; h Department of Psychosis Studies, Institute of Psychiatry, Psychology & Neuroscience, King's College London, London, UK; i Department of Neuroimaging, Institute of Psychiatry, Psychology & Neuroscience, King's College London, London, UK

**Keywords:** Bipolar disorder type I, First-degree relative, Cortical thickness, Endophenotype, Surface area

## Abstract

**Objectives:**

So far, few studies have investigated cortical thickness (CT) and surface area (SA) measures in bipolar disorder type I (BDI) in comparison to a high genetic risk group such as first-degree relatives (FR). This study aimed to examine CT and SA differences between BDI, FR and healthy controls (HC).

**Methods:**

3D T1 magnetic resonance images were acquired from 27 euthymic BDI patients, 24 unaffected FR and 29 HC. CT and SA measures were obtained with FreeSurfer version 5.3.0. Generalized estimating equations were used to compare CT and SA between groups. Group comparisons were repeated with restricting the FR group to 17 siblings (FR-SB) only.

**Results:**

\Mean age in years was 36.3 ± 9.5 for BDI, 32.1 ± 10.9 for FR, 34.7 ± 9.8 for FR-SB and 33.1 ± 9.0 for HC group respectively. BDI patients revealed larger SA of left pars triangularis (LPT) compared to HC (*p* = .001). In addition, increased SA in superior temporal cortex (STC) in FR-SB group compared to HC was identified (*p* = .0001).

**Conclusions:**

Our result of increased SA in LPT of BDI could be a disease marker and increased SA in STC of FR-SB could be a marker related with resilience to illness.

## Highlights

• Increased surface area of left pars triangularis in bipolar disorder type I patients could be a disease marker.
• Increased surface area of superior temporal cortex could be a resilience marker for bipolar disorder type I.
• More studies should be conducted on the morphometric characteristics of cortical surface area measures.

## 1 Introduction

Bipolar disorder (BD) is characterized by episodes of mania, hypomania and depression and the wellbeing state between the episodes called euthymia (American Psychiatric Association, 2000). The lifetime prevalence is 0.6% for BD type I (BDI), 0.4% for BD type II and extends to 2.4% considering the whole spectrum of BD (Merikangas et al., 2011).

BD is arguably one of the most heritable of the Axis I disorders with a heritability estimate to be as high as 93% (Kieseppa et al., 2004). One way to investigate vulnerability factors for highly heritable diseases like BD is an endophenotype based approach (Hasler et al., 2006). An endophenotype is a biomarker, which is heritable, associated with illness, state-independent, co-segregates with illness in families and is found at a higher rate in non-effected family members compared to general population (Gottesman and Gould, 2003). A wide variety of endophenotypes have been proposed in the psychiatric literature and advanced tools of neuroimaging such as structural magnetic resonance imaging (sMRI) promise to expand these further (Gottesman and Gould, 2003).

sMRI in BD has been the focus of attention for >20 years with the majority of studies in the literature examining gray matter volume. So far, the most consistent findings in BD patients are reduced volume in insula (Bora et al., 2010; Ellison-Wright and Bullmore, 2010; Selvaraj et al., 2012), anterior cingulate cortex (ACC) (Bora et al., 2010; Ellison-Wright and Bullmore, 2010), inferior frontal cortex (IFC) (Houenou et al., 2011; Selvaraj et al., 2012) and enlargement in lateral ventricles (Arnone et al., 2009; Kempton et al., 2008) compared to healthy controls (HC). Gray matter volume changes in the relatives of BD patients are inconclusive but there are few replicated findings which includes increased volume in IFC, amygdala, caudate, parahippocampal cortex and decreased volume in orbitofrontal cortex (OFC), insula and cerebellum compared to HC (Nery et al., 2013).

Gray matter volume is a function of two different morphometric structures called as cortical thickness (CT) and surface area (SA) (Hanford et al., 2016a; Winkler et al., 2010). While SA is reflective of number of columns, CT measures reflect the number of cells within each column (Hanford et al., 2016a). CT and SA are both heritable, globally and regionally independent and genetically uncorrelated (Sugranyes et al., 2017). It has been argued that these indices should be studied separately and may be preferred over gray matter volume in terms of brain imaging endophenotype (Winkler et al., 2010). Recent advances in neuroimaging analysis such as surface based techniques enable researchers to measure these two different cortical indices accurately (Winkler et al., 2010).

To date, studies on CT in BD patients have revealed some consistent results including cortical thinning in ACC, OFC, paracingulate, dorsolateral, ventrolateral, superior frontal and superior temporal cortex (STC) compared to HC (Hanford et al., 2016a). In terms of SA in BD; although a number of studies did not find significant results (Elvsashagen et al., 2013; Fornito et al., 2008; Janssen et al., 2014; Rimol et al., 2012; Roberts et al., 2016), other studies reported increased SA in STC, precuneus, insula, temporal pole, supramarginal, postcentral and superior frontal cortex and decreased SA in frontotemporal cortices and posterior cingulate cortex in BD patients compared to HC (Abe et al., 2016; Fung et al., 2015; Hartberg et al., 2011). In addition to these small sample sized studies on cortical abnormalities in BD, a mega analysis by ENIGMA consortium, which was comprised of 2447 BD patients and 4056 HC, was conducted recently. This largest study to date showed decreased CT in pars opercularis, fusiform and rostral middle frontal cortex in BD patients compared to HC but didn't show any differences in SA between groups (Hibar et al., 2018).

Regarding CT and SA in relatives of BD patients, 6 studies have been conducted. Two heritability studies revealed that cortical thickening in supramarginal cortex and rolandic operculum, SA expansion in supramarginal, inferior parietal and posterior cingulate cortex and cortical thinning in STC, IFC, OFC, inferior temporal, fusiform and lingual cortex are associated with genetic liability to develop BD in unaffected relatives (Bootsman et al., 2015; Fears et al., 2014). The rest four studies showed cortical thinning in IFC, STC, parahippocampal, middle and inferior temporal, middle frontal, fusiform and supramarginal cortex, cortical thickening in postcentral cortex (Hanford et al., 2016b; Papmeyer et al., 2015; Roberts et al., 2016) and no differences in CT or SA (Roberts et al., 2016; Sugranyes et al., 2017) in the relatives of BD compared to HC.

We recently reported gray matter volume results from the same cohort with a significant main effect of the group in cerebellum, IFC, parahippocampal, lingual, posterior cingulate and supramarginal cortex. Larger IFC and smaller cerebellum were demonstrated in both BDI and FR (Saricicek et al., 2015). In the present study, we primarily aimed to investigate gray matter volume determinants, CT and SA, between BDI patients, their FR and HC. Based on the studies in the literature and our previous findings, ACC and IFC were considered as strong candidates to be a BDI endophenotype and were selected as regions of interest (ROIs) for this study. We also aimed to conduct an exploratory analysis of SA and CT of other brain regions apart from the defined ROIs between groups to provide information for future research. Considering the summarized literature above, we hypothesized that BD and FR will have decreased CT and/or SA of the defined ROIs and will differ from HC.

## 2 Methods and materials

### 2.1 Participants

This study was approved by Dokuz Eylul University Hospital Ethics Committee and was conducted between January 2012 and January 2014 in accordance with the latest version of Declaration of Helsinki. All participants provided written informed consent. BDI patients were recruited from Bipolar Disorder Outpatient Unit, Department of Psychiatry, Dokuz Eylul University, where they had been receiving their monthly follow-up care. For the recruitment of the relative group, unaffected FRs of the enrolled patients were first approached for participation. Nevertheless, due to either lack or unavailability of an unaffected FR for each study patient, FRs of non-study patients were also recruited through advertisements at inpatient and outpatient unit. HCs were recruited through advertisements at the university hospital and on medical school campus. The inclusion criteria for patient group were having a diagnosis of BDI according to DSM-IV, aging between 18 and 65 years, being in euthymic state (according to DSM-IV and scoring ≤7 on both Young Mania Rating Scale and Hamilton Rating Scale for Depression) for at least six months and having no axis I comorbidity. The inclusion criteria for FR were having no lifetime axis I diagnosis, and for HC no personal or family history for psychiatric disorders at the time of recruitment. The following exclusion criteria were applied to all groups: presence of auditory or visual impairment, history of neurosurgical intervention, being pregnant or breastfeeding, diagnosis of neurocognitive illness or substance use during the preceding six weeks before participating in the study. All participants were evaluated using the Structured Clinical Interview for Diagnostic Statistical Manual-IV (DSM-IV) (SCID-I). During the study period, forty-eight BDI patients, 35 unaffected FR, and 44 HC were assessed for inclusion. The following participants were excluded: 11 BDI patients were not euthymic at the time of the assessment; 7 BDI patients, 7 FR and 14 HC decided to withdraw from the study before MRI scanning; and 3 BDI patients, 4 FR and 1 HC were excluded for movement artifacts after MRI scanning. Thus, the final sample included 27 BDI patients, 24 FR (17 siblings, 6 offspring, 1 parent) and 29 HC.

### 2.2 MRI acquisition and preprocessing

MRI data was acquired using a 1.5 T Philips Tesla Achieva Magnetic Resonance Imaging Scanner and SENSE head coil/8 channel in the Department of Radiology, Dokuz Eylul University. 3D T1-fast field echo (FFE) axial images (repetition time (TR) =8.7 ms, echo time (TE) =4 ms, flip angle = 8 ^o^, field of view (FOV) =230 mm × 220 mm, slice thickness = 1 mm, number of signal averages (NSA) =1, matrix = 192) were used for surface-based analysis. In addition, axial T2 weighted turbo spin echo and proton density images (TR = 3000 ms, TE = 96 ms, flip angle = 90, FOV = 240 mm × 220 mm, slice thickness = 3 mm NSA = 1, Matrix = 256) were acquired for neuroradiological assessment.

CT and SA measures were obtained with the FreeSurfer version 5.3.0 image analysis software (http://surfer.nmr.mgh.harvard.edu/). The Freesurfer automated pipeline starts with motion and intensity correction and is followed by Talairach registration, normalization, skull stripping and subcortical segmentation. After subcortical segmentation, an initial surface by tilling the gray-white matter boundary (white matter surface) and a second surface by tracing the gray-CSF boundary (the pial surface) are generated. Following this, surfaces are inflated for visualization of the sulci and the topological defects are corrected with automatic topology fixing. The pipeline ends with surface registration to the spherical atlas, cortical parcellation and labelling. The distance between equivalent vertices of white matter surface and pial surface gives the CT and the sum of the area of the vertices on white matter surface gives the SA (Dale et al., 1999; Desikan et al., 2006; Fischl and Dale, 2000; Fischl et al., 1999).

The Desikan-Killiany atlas was used for cortical parcellation and labelling which produces 34 regions per hemisphere. In terms of representations of the study ROIs in the Desikan-Killiany atlas; ACC is represented by a rostral and caudal region and the IFC is represented by three regions, the pars orbitalis, triangularis and opercularis.

Standardized quality protocol for the analysis of FreeSurfer mean CT and SA ROI data developed by the ENIGMA consortium was used for quality checking (QC) of the preprocessed images. This protocol uses R and Matlab scripts and Quality Assurance Tools for FreeSurfer and consists of three steps, namely, outlier detection, internal QC and external QC. Outlier detection creates a file which reveals the subjects that are outliers, and for which structures they are outliers for to make sure that these subjects inspected closely as QC proceed. In internal QC step, snapshots of preprocessed images from different brain sections are produced for each subject to visually inspect internal cortical segmentation for under/overestimations. In external QC, 4 images with external views of the segmentations from different angles are produced for visually checking cortical labels and anatomical boundaries and for confirming errors spotted on internal QC if there is any (http://enigma.ini.usc.edu/protocols/imaging-protocols). All preprocessed images in this study were subjected to these three steps and showed sufficient quality to be included in further analysis.

### 2.3 Statistical Analysis

All statistical analyses were carried out using Statistical Package for Social Science (SPSS Inc., v23). Comparisons of sociodemographic variables between groups were performed using chi-square test or analysis of variance.

For each subject, average CT and SA measures for each brain region were extracted to SPSS. To compare CT and SA measures among three groups (BDI/FR/HC), generalized estimating equations (GEE) models were used to accommodate within-subject dependencies arising from the inclusion of participants from the same family. GEE is a statistical analysis that extends the generalized linear models to allow for analysis of repeated measures or other correlated observations such as clustered data. It assumes that cases are dependent within subjects and independent between subjects and estimates a correlation matrix that represents the within subject dependencies as a part of the model (IBM, 2015). For each GEE model where CT was the dependent variable, we statistically controlled for age and gender and where SA was the dependent variable, we controlled for age, gender and total intracranial volume (Barnes et al., 2010). The same analyses were repeated by restricting the FR group to siblings only (FR-SB) to constitute a more homogenous group in terms of genetic risk for BDI.

Where there was a significant difference of BDI group compared to HC on CT or SA measures, the effect of clinical variables, namely duration of illness, total number of episodes, episode frequency, current lithium, atypical antipsychotic and valproic acid use were also examined using univariate general linear models (UGLMs). For each UGLM where CT was the dependent variable, we statistically controlled for age and gender and where SA was the dependent variable, we controlled for age, gender and total intracranial volume (Barnes et al., 2010)

Corrections for multiple testing were carried out using bonferroni correction. (McDonald, 2014). For ROI analysis, corrections were applied to the 10 tests (5 brain regions per each hemisphere) separately for CT and SA and the statistical significance level was set to *p* ≤ .005 (α/10). For exploratory analysis, corrections were applied to 58 tests (29 brain regions per each hemisphere) separately for CT and SA and the statistical significance level was set to *p* ≤ .0008 (α/58). For clinical variables, corrections were applied to 6 tests and the statistical significance level was set to *p* ≤ .008 (α/6). We presented the uncorrected *p* values throughout and only implied the findings as significant if the p values survived the Bonferroni correction as being equal or lower than the already set statistical significance level for each analysis mentioned above.

## 3 Results

Demographic characteristics of the groups and the clinical variables for the BDI group are shown in Table 1, Table 2. There were no differences between groups in any of the demographic variables.

**Table 1:** Demographic characteristics of the groups.

					BD vs FR vs HC		BD vs FR-SB vs HC	
	BDI (n = 27)	FR (n = 24)	FR-SB (n = 17)	HC (n = 29)	Test-value	p-Value	Test-value	p-Value
Age in years (mean ± SD)	36.3 ± 9.5	32.1 ± 10.9	34.7 ± 9.8	33.1 ± 9.0	F = 1.31	0.28	F = 0.83	0.44
Gender: Female	63% (n = 17)	54% (*n* = 13)	47.1% (*n* = 8)	62% (*n* = 18)	χ2 = 0.49	0.78	χ2 = 1.29	0.52
Education in years (mean ± SD)	12.9 ± 2.8	11.5 ± 2.9	11.4 ± 2.9	13.6 ± 3.8	F = 2.90	0.061	F = 2.35	0.10
Handedness: Right	96% (*n* = 26)	96% (*n* = 23)	100% (n = 17)	97% (*n* = 28)	χ2 = 0.45	1	χ2 = 0.79	1
FR distribution		71% siblings (n = 17)						
		25% offspring (*n* = 6)						
		4% parent (*n* = 1)						

BDI: bipolar disorder type I, FR: first degree relatives, FR-SB: siblings, HC: healthy controls.

**Table 2:** Clinical characteristics of BDI group.

Clinical variables	BDI (n = 27)
Duration of illness in years (mean ± SD)	10.25 ± 6.70
Age of illness onset in years (mean ± SD)	26.04 ± 8.18
Total number of manic/hypomanic/mixed episodes (mean ± SD)	2.96 ± 2.12
Total number of depressive episodes (mean ± SD)	1.78 ± 1.67
Total number of episodes (mean ± SD)	4.74 ± 2.99
Episode frequency (mean ± SD)	0.5 ± 0.2
Current lithium use (%, n)	66.7%, 18
Current valproic acid use (%, n)	40.7%, 11
Current antipsychotic use (atypical),(%, n)	59.3%, 16

BDI: bipolar disorder type I.

### 3.1 ROI Analysis of ACC and IFC

There were no significant group differences in CT of ACC and IFC between BDI, FR and HC (Table 3). With regard to SA; the BDI group was significantly different from HC in the left pars triangularis (LPT) (B = 129.45, %95 CI = (50.12, 208.79), *p* = .001) (Table 3).

**Table 3:** Comparison of CT and SA measurement of the ROIs between groups.

						BD vs FR vs HC		BD vs FR-SB vs HC	
						p-Values		p-Values	
	Brain Regions	BD (n = 27)	FR (n = 24)	FR-SB (n = 17)	HC (n = 29)	BD vs HC	FR vs HC	BD vs HC	FR-SB vs HC
CT (mm)	L Caudal anterior cingulate	2.52 ± 0.24	2.63 ± 0.24	2.68 ± 0.24	2.57 ± 0.22	0.609	0.309	0.648	0.069
	L Rostral anterior cingulate	2.77 ± 0.20	2.89 ± 0.21	2.90 ± 0.22	2.90 ± 0.29	0.084	0.924	0.093	0.681
	L Pars opercularis	2.51 ± 0.11	2.54 ± 0.14	2.53 ± 0.13	2.53 ± 0.17	0.818	0.976	0.839	0.785
	L Pars orbitalis	2.59 ± 0.21	2.59 ± 0.25	2.58 ± 0.26	2.58 ± 0.22	0.555	0.944	0.51	0.674
	L Pars triangularis	2.38 ± 0.14	2.41 ± 0.92	2.41 ± 0.10	2.41 ± 0.17	0.904	0.939	0.973	0.695
	R Caudal anterior cingulate	2.45 ± 0.18	2.51 ± 0.29	2.46 ± 0.26	2.46 ± 0.22	0.891	0.404	0.905	0.663
	R Rostral anterior cingulate	2.67 ± 0.22	2.74 ± 0.19	2.75 ± 0.18	2.82 ± 0.20	0.026	0.11	0.028	0.365
	R Pars opercularis	2.45 ± 0.14	2.49 ± 0.15	2.47 ± 0.14	2.54 ± 0.19	0.095	0.281	0.085	0.302
	R Pars orbitalis	2.60 ± 0.22	2.66 ± 0.23	2.62 ± 0.23	2.65 ± 0.25	0.797	0.83	0.795	0.832
	R Pars triangularis	2.38 ± 0.17	2.42 ± 0.14	2.42 ± 0.14	2.45 ± 0.17	0.231	0.401	0.252	0.799
SA (mm^2^)	L Caudal anterior cingulate	664.74 ± 134.40	626.25 ± 107.10	630.00 ± 112.94	644.20 ± 128.01	0.576	0.488	0.625	0.577
	L Rostral anterior cingulate	842.07 ± 122.58	772.75 ± 115.92	768.05 ± 99.26	789.79 ± 132.29	0.063	0.38	0.069	0.381
	L Pars opercularis	1548.03 ± 207.66	1613.75 ± 240.30	1618.05 ± 236.83	1506.86 ± 217.79	0.316	0.127	0.233	0.072
	L Pars orbitalis	616.51 ± 75.80	626.29 ± 85.26	622.58 ± 89.31	602.75 ± 74.29	0.307	0.445	0.336	0.501
	***L Pars triangularis***	***1266.63 ± 179.42***	***1184.75 ± 170.92***	***1209.11 ± 151.71***	***1148.03 ± 177.48***	***0.001***	0.735	***0.0003***	0.267
	R Caudal anterior cingulate	761.92 ± 155.54	748.95 ± 143.68	749.52 ± 153.06	758.55 ± 158.54	0.833	0.607	0.804	0.676
	R Rostral anterior cingulate	677.92 ± 137.65	625.50 ± 116.58	610.29 ± 116.12	659.96 ± 96.61	0.495	0.143	0.506	0.078
	R Pars opercularis	1331.14 ± 197.39	1303.41 ± 126.05	1301.58 ± 136.18	1284.37 ± 231.68	0.195	0.96	0.163	0.899
	R Pars orbitalis	763.59 ± 101.67	786.50 ± 64.85	785.82 ± 68.28	731.62 ± 110.32	0.108	0.065	0.112	0.104
	R Pars triangularis	1433.44 ± 266.49	1388.66 ± 206.83	1391.52 ± 219.39	1359.34 ± 251.24	0.153	0.941	0.128	0.83

BDI: bipolar disorder type I, FR: first degree relatives, FR-SB: siblings; HC: healthy controls, CT: cortical thickness, SA: surface area, ROI: region of interest.

Statistically significant results that were survived Bonferroni correction for multiple comparisons (p ≤ 0.005) are highlighted in bold and italics.

Repeating the analysis with the FR-SB revealed similar results in terms of CT. Regarding SA, the same significant difference was established in the LPT in BDI group compared to HC (B = 133.09, %95 CI = (59.74, 206.43), *p* = .0003) (Table 3).

### 3.2 Exploratory analysis of other brain regions

BDI, FR and HC comparisons revealed no significant differences in CT or SA in the exploratory analysis (S1, S2). In terms of BDI, FR-SB and HC comparisons; there were no significant differences in CT measures of the other brain regions (S3). Regarding SA measures, FR-SB showed significant difference in SA of right STC compared to HC (B = 291.30, %95 CI = (141.01, 441.60) *p* = .0001) (S4).

### 3.3 Relationship between clinical variables and LPT SA

There was no significant relationship between duration of illness (*p* = .15), total number of episodes (*p* = .97), episode frequency (*p* = .06), current lithium (*p* = .09), atypical antipsychotic (*p* = .44) or valproic acid use (p = .15) and SA of LPT in BDI group (S5).

## 4 Discussion

In this study, we examined CT and SA abnormalities of ACC and IFC in patients with BDI, their FR/FR-SB and HC. We also carried out an exploratory analysis on other brain regions for SA and CT differences between groups. For our hypothesized regions, we showed larger SA of LPT in BDI group compared to HC. There were no significant relationships between SA of LPT and clinical variables in BDI patients. Our exploratory analysis revealed larger SA of STC in FR-SB compared to HC.

Pars triangularis (Broadmann Area (BA) 45) is one of the subparts of IFC that has a crucial role in semantic processing, memory and executive functions (Clark et al., 2010) which were shown to be impaired in BD patients with middle to large effect sizes (Bora et al., 2009). Most of the data considering IFC in BD including the ENIGMA data showed decreased volume or cortical thinning in this area of interest (Hanford et al., 2016a; Hibar et al., 2018; Houenou et al., 2011; Selvaraj et al., 2012). On the other hand, there are also studies including our previous publication that revealed larger volumes in IFC in BD patients compared to HC (Hajek et al., 2013; Saricicek et al., 2015). Regarding SA; there is only one study that revealed smaller SA of IFC in BD compared to HC (Abe et al., 2016).

Our finding of enlarged SA of LPT in BDI patients is in contrast with the previous studies and our primary hypothesis. One explanation for this finding could be the particular clinical characteristics of our sample. Previous studies showed that decreased volume in IFC in BD is associated with burden of illness, minimal lifetime exposure to lithium, being multi-episode rather than being first episode and increased incidence of manic episodes during clinical course (Abe et al., 2015; Hajek et al., 2013; Matsuo et al., 2012). Our sample consisted of patients who were euthymic for a long period of time (approximately two years), had shorter periods of mood episodes throughout the illness course and were primarily on lithium at the time of the study or had used lithium previously. Considering these clinical characteristics of our patient sample, our finding of an enlarged SA in LPT may be arisen from the joint effects of better disease course and long-term lithium use. Nevertheless, in contrary to this interpretation, we couldn't show any association between SA of LPT and clinical variables in BDI group. One possible explanation for the negative findings in treatment variables could be the past exposure to these medications or the dose and duration of previous and current medication use of patients, which weren't taken into account in our study (Hafeman et al., 2012). In addition, for each medication group, comparisons were made between the patients who were taking the relevant medication with the ones who were not at the time of study entry. However, most of our patients were on different combinations of lithium, valproic acid and atypical antipsychotics and different medications might interact to produce effects that are different to those of each medication alone (Hafeman et al., 2012). On the other hand, our non-significant finding in total number of episodes and episode frequency may have arisen from not examining the manic and depressive episodes separately as they were shown to have different effects on gray matter (Abe et al., 2015; Lisy et al., 2011).

From another aspect; SA measures are obtained from gray-white matter boundary and it is speculated that SA expansion might reflect not only the gray matter but also the pathology of underlying white matter fibers as more tension or shrinkage of these fibers could lead to deeper sulci and extended cortical SA (Van Essen, 1997). This speculation is supported with our diffusion tensor imaging findings in the same cohort, which revealed decreased fractional anisotropy in several white matter tracts including uncinate and inferior fronto-occipital fasciculus that are known to be closely related with IFC in BDI patients compared to controls (Saricicek et al., 2016).

When examining IFC SA of FR/FR-SB; our finding of no difference in any subparts of IFC compared to HC group is in line with numerous previous studies that measured gray matter volume, CT or SA in FR of BD patients (Hanford et al., 2016b; Nery et al., 2013; Papmeyer et al., 2015; Roberts et al., 2016). However, it should be noted that there are also studies that reported increased gray matter volume or decreased CT in IFC in FR of BD patients (Hajek et al., 2013; Saricicek et al., 2015). As we found no differences in FR or FR-SB compared to HC, we represent our finding of increased SA of LPT in BDI as a disease marker rather than an endophenotype for BDI.

In contrast to our primary hypothesis, there were no significant differences in the CT or SA of ACC in BDI or FR/FR-SB compared to healthy controls. ACC (BA 24,25,32,33) is a highly connected region with limbic and prefrontal brain structures and it is involved in execution of behavior, shifting attention, emotion and memory functions of the brain (Clark et al., 2010). Although meta-analyses on volume and the majority of CT studies reported reduced gray matter measures (Bora et al., 2010; Hanford et al., 2016a; Houenou et al., 2011) in ACC of BD patients compared to controls; our result is compatible with all studies examining SA (Elvsashagen et al., 2013; Fung et al., 2015; Hartberg et al., 2011; Janssen et al., 2014; Rimol et al., 2012) and a few studies of CT or volume, which failed to show any significant differences in this area of interest (Rimol et al., 2010; Zimmerman et al., 2006). One reason of our non-significant finding in BDI patients could be related to current or previous lithium use of the majority of our patient group as it has been associated with increased gray matter volumes in ACC (Hafeman et al., 2012). Another reason could be that we may have been underpowered to detect any gray matter change due to the relatively small sample size of this study. At the time of the analysis plan, a power calculation for ACC was performed using the results of a meta-analysis on gray matter volume changes in BD that revealed an effect size d of 1.17 for this area of interest (Kempton et al., 2008) as there were only a few papers on CT and SA in BD with small sample sizes. After converting the effect size d = 1.17 of the ACC to f = 0.585, this power calculation (alpha = 0.05, power = 0.8) indicated a minimum total sample size of 32. On the other hand, a mega-analysis on CT and SA in BD (Hibar et al., 2018) compared to HC was published after the data collection and analysis were completed for this study. This mega-analysis enabled us to perform a second power calculation using an effect size for CT and SA of ACC (rather than volume) in BD which was the scope of this study. The largest effect size d that the mega-analysis reported for CT and SA measures of ACC subparts was used for the second power calculation, which was 0.153 for the CT of left rostral ACC in BD. After converting effect size d of 0.153 to f as 0.0765, this power calculation (alpha = 0.05, power = 0.8) revealed that the minimum total sample size needed is 1650. To conclude, although our sample size was appropriately powered to show differences in gray matter volume measures, it was clearly underpowered to detect any differences in CT and SA between groups.

In terms of FR of BD; our study is in line with the majority of the studies on volume, CT or SA, which failed to show any significant differences in ACC in relatives of BD compared to HC (Hanford et al., 2016b; Nery et al., 2013; Papmeyer et al., 2015; Roberts et al., 2016; Sugranyes et al., 2017). Overall, despite our non-significant result in this area, ACC seems to be a marker related with disease but not a candidate to be an endophenotype. We propose that ACC should continue to be a region of interest for BD and be studied in larger drug-free patient groups.

Our exploratory analysis revealed larger SA in FR-SB compared to HC. STC is known for its role in processing auditory stimuli, language, speech and communicative gestures (Clark et al., 2010). Looking at the literature regarding STC in BD patients, most of the volume and CT studies showed gray matter decrease in this area of interest (Hanford et al., 2016a; Li et al., 2011; Takahashi et al., 2010). On the other hand, there are also studies that reported increased volume and SA in BD compared to HC (Adler et al., 2007; Fung et al., 2015). In regard to FR of BD, the majority of the studies on volume, CT or SA were not able to find any differences in STC compared to HC (Hanford et al., 2016b; Nery et al., 2013; Papmeyer et al., 2015; Roberts et al., 2016; Sugranyes et al., 2017). Taken together the current literature and our non-significant finding between BDI and HC, our finding of the enlargement of STC in FR-SB may be a resilience factor and needs to be further investigated in the future studies.

Comparing the results of this study with our volumetric analysis of the same cohort, the only overlap was the enlargement of LPT in BDI patients compared to controls. A comprehensive comparison of measuring gray matter with different analysis techniques is beyond the scope of this study but possible reasons will be briefly discussed. First of all, CT, SA and volume measures follow different neurodevelopmental trajectories both in pattern and timing and should be evaluated independently (Panizzon et al., 2009). Second, volumetric measures may be partially mediated by cortical measures and this discrepancy may have derived from local gyrification index, a third surface measure which contributes to gray matter volume and was not measured in this study (Palaniyappan and Liddle, 2012). Apart of that, this difference may be related with minor gray matter changes in CT and SA that can't be detected with current analysis techniques but becoming apparent as a cumulative manner in volume measures. From another point of view, this discrepancy could be the result of different statistical procedures undertaken in each study. In our volume analysis, the group effect was controlled for total intracranial volume and years of education, and the multiple comparison correction was carried out with Monte-Carlo Stimulation. In this study, we controlled group effect with different variables including age, gender, total intracranial volume and Bonferroni corrections were carried throughout. Furthermore, different softwares were used as Statistical Parametric Mapping for volume and FreeSurfer for CT and SA analysis. This could be the other reason for the different results as these softwares follow different techniques in segmentation and spatial normalization (Katuwal et al., 2016).

Our study has several limitations. First, small sample sizes in each group could have increased the possibility of type II error and it makes our results difficult to generalize to the wider patient population. Second, our results of FR group should be evaluated with the information that siblings, parents and offspring have different genetic risk for BD. Furthermore, it has been shown that approximately the 45% of bipolar disorder patients have their illness onset before the mean age of 21 and up to 80% before the mean age of 35 (Geoffroy et al., 2013).The mean age of our FR group was 32 and FR-SB was 34, which were very near to exceed the defined age threshold for the onset of illness. From this aspect, our relative sample could be at low risk or resilient for developing BDI which may have hindered finding any potential brain areas as a risk marker.

In conclusion, our result of increased SA in LPT of BDI compared to HC could be a disease marker and increased SA in STC of FR-SB compared to HC could be a marker related with resilience. Longitudinal studies are needed in BD patients and high-risk groups to clarify the discrepancies between cross-sectional studies and more studies should be conducted on the morphometric characteristics of cortical SA.

## Appendix A Supplementary data

Supplementary material Image 1
