# Supplementary material for: Cortical thickness and surface area as an endophenotype in bipolar disorder type I patients and their first-degree relatives
Source: Neuroimage Clin. 2019 Jan 29;22:101695. doi: 10.1016/j.nicl.2019.101695 (PMC6370861; doi:10.1016/j.nicl.2019.101695)
Supplement: Supplementary file 1 — Supplementary material [file mmc1.docx]

**SUPPLEMENTARY MATERIAL**

**S1.** **Comparison of CT measurement of the other brain regions between BDI, FR and HC**

*(BDI: bipolar disorder type I, FR: first degree relatives, HC: healthy controls, CT:cortical thickness)*

| ***Brain Regions (CT)*** | ***Hemisphere*** | ***BDI***  ***(n=27)*** | ***FR (n=24)*** | ***HC***  ***(n=29)*** | ***BD vs HC***  ***p values*** | ***FR vs HC***  ***p values*** |
| --- | --- | --- | --- | --- | --- | --- |
| Bankssts | L | 2.47±0.15 | 2.51±0.19 | 2.47±0.19 | 0.648 | 0.589 |
|  | R | 2.56±0.16 | 2.55±0.20 | 2.50±0.21 | 0.189 | 0.298 |
| Caudal middle frontal | L | 2.46±0.14 | 2.49±0.20 | 2.48±0.17 | 0.928 | 0.934 |
|  | R | 2.42±0.14 | 2.47±0.17 | 2.47±0.19 | 0.452 | 0.875 |
| Cuneus | L | 1.73±0.14 | 1.70±0.11 | 1.73±0.16 | 0.969 | 0.342 |
|  | R | 1.70±0.12 | 1.76±0.11 | 1.75±0.20 | 0.299 | 0.838 |
| Entorhinal | L | 3.34±0.31 | 3.35±0.36 | 3.43±0.29 | 0.331 | 0.448 |
|  | R | 3.57±0.32 | 3.59±0.44 | 3.55±0.40 | 0.783 | 0.686 |
| Fusiform | L | 2.60±0.15 | 2.64±0.16 | 2.65±0.15 | 0.329 | 0.932 |
|  | R | 2.66±0.13 | 2.66±0.16 | 2.70±0.19 | 0.49 | 0.402 |
| Inferior parietal | L | 2.40±0.11 | 2.43±0.15 | 2.45±0.14 | 0.402 | 0.501 |
|  | R | 2.42±0.12 | 2.47±0.13 | 2.46±0.16 | 0.567 | 0.931 |
| Inferior temporal | L | 2.72±0.13 | 2.75±0.19 | 2.76±0.15 | 0.608 | 0.759 |
|  | R | 2.81±0.17 | 2.87±0.17 | 2.88±0.15 | 0.18 | 0.845 |
| Isthmus cingulate | L | 2.32±0.19 | 2.38±0.17 | 2.36±0.17 | 0.664 | 0.819 |
|  | R | 2.22±0.19 | 2.22±0.17 | 2.34±0.18 | 0.051 | 0.009 |
| Lateral occipital | L | 2.08±0.12 | 2.15±0.12 | 2.12±0.17 | 0.448 | 0.628 |
|  | R | 2.15±0.14 | 2.14±0.15 | 2.21±0.16 | 0.256 | 0.091 |
| Lateral orbitofrontal | L | 2.53±0.17 | 2.57±0.22 | 2.58±0.16 | 0.67 | 0.748 |
|  | R | 2.50±0.16 | 2.53±0.13 | 2.52±0.14 | 0.923 | 0.921 |
| Lingual | L | 1.88±0.15 | 1.93±0.12 | 1.96±0.17 | 0.088 | 0.415 |
|  | R | 1.91±0.12 | 1.93±0.11 | 1.99±0.15 | 0.052 | 0.137 |
| Medial orbitofrontal | L | 2.43±0.13 | 2.45±0.16 | 2.44±0.17 | 0.744 | 0.915 |
|  | R | 2.33±0.17 | 2.39±0.17 | 2.40±0.19 | 0.341 | 0.809 |
| Middle temporal | L | 2.82±0.16 | 2.84±0.15 | 2.85±0.13 | 0.679 | 0.775 |
|  | R | 2.84±0.12 | 2.88±0.18 | 2.90±0.18 | 0.229 | 0.596 |
| Parahippocampal | L | 2.69±0.30 | 2.65±0.27 | 2.72±0.23 | 0.728 | 0.381 |
|  | R | 2.66±0.23 | 2.72±0.28 | 2.74±0.21 | 0.256 | 0.651 |
| Paracentral | L | 2.25±0.13 | 2.31±0.17 | 2.30±0.17 | 0.354 | 0.819 |
|  | R | 2.29±0.15 | 2.33±0.19 | 2.32±0.16 | 0.758 | 0.849 |
| Pericalcarine | L | 1.46±0.09 | 1.47±0.11 | 1.54±0.31 | 0.175 | 0.253 |
|  | R | 1.48±0.11 | 1.50±0.10 | 1.56±0.27 | 0.093 | 0.229 |
| Postcentral | L | 2.00±0.11 | 2.01±0.12 | 2.00±0.14 | 0.81 | 0.685 |
|  | R | 1.97±0.11 | 1.98±0.11 | 1.99±0.13 | 0.881 | 0.794 |
| Posterior cingulate | L | 2.38±0.11 | 2.40±0.14 | 2.44±0.16 | 0.362 | 0.238 |
|  | R | 2.36±0.15 | 2.38±0.15 | 2.40±0.14 | 0.589 | 0.568 |
| Precentral | L | 2.45±0.11 | 2.46±0.17 | 2.47±0.18 | 0.878 | 0.771 |
|  | R | 2.43±0.11 | 2.45±0.17 | 2.46±0.18 | 0.822 | 0.822 |
| Precuneus | L | 2.25±0.14 | 2.26±0.13 | 2.28±0.14 | 0.603 | 0.529 |
|  | R | 2.24±0.14 | 2.27±0.13 | 2.28±0.14 | 0.405 | 0.758 |
| Rostral middle frontal | L | 2.28±0.14 | 2.36±0.13 | 2.37±0.15 | 0.038 | 0.652 |
|  | R | 2.24±0.12 | 2.27±0.10 | 2.33±0.15 | 0.041 | 0.059 |
| Superior frontal | L | 2.67±0.14 | 2.71±0.19 | 2.73±0.19 | 0.436 | 0.711 |
|  | R | 2.24±0.12 | 2.27±0.10 | 2.33±0.15 | 0.629 | 0.8 |
| Superior parietal | L | 2.11±0.12 | 2.14±0.12 | 2.14±0.17 | 0.614 | 0.954 |
|  | R | 2.10±0.11 | 2.12±0.12 | 2.13±0.14 | 0.638 | 0.799 |
| Superior temporal | L | 2.70±0.15 | 2.69±0.18 | 2.73±0.17 | 0.823 | 0.312 |
|  | R | 2.77±0.14 | 2.77±0.20 | 2.77±0.21 | 0.602 | 0.886 |
| Supramarginal | L | 2.48±0.12 | 2.51±0.15 | 2.51±0.16 | 0.811 | 0.93 |
|  | R | 2.45±0.11 | 2.50±0.15 | 2.51±0.15 | 0.368 | 0.749 |
| Frontal pole | L | 2.75±0.25 | 2.80±0.36 | 2.83±0.29 | 0.603 | 0.714 |
|  | R | 2.72±0.28 | 2.76±0.29 | 2.87±0.32 | 0.08 | 0.177 |
| Temporal pole | L | 3.52±0.26 | 3.60±0.32 | 3.64±0.32 | 0.255 | 0.553 |
|  | R | 3.73±0.30 | 3.70±0.43 | 3.77±0.37 | 0.781 | 0.416 |
| Transverse temporal | L | 2.30±0.20 | 2.22±0.21 | 2.30±0.23 | 0.867 | 0.156 |
|  | R | 2.40±0.13 | 2.31±0.28 | 2.39±0.19 | 0.662 | 0.115 |
| Insula | L | 2.97±0.09 | 3.02±0.16 | 3.01±0.15 | 0.711 | 0.934 |
|  | R | 2.91±0.13 | 2.96±0.17 | 2.95±0.16 | 0.732 | 0.994 |

**S2.** **Comparison of SA measurement of the other brain regions between BDI, FR and HC**

*(BDI: bipolar disorder type I, FR: first degree relatives, HC: healthy controls, SA:surface area)*

| ***Brain Regions (SA)*** | ***Hemisphere*** | ***BDI (n=27)*** | ***FR***  ***(n=24)*** | ***HC (n=29)*** | ***BD vs HC***  ***p values*** | ***FR vs HC***  ***p values*** |
| --- | --- | --- | --- | --- | --- | --- |
| Bankssts | L | 1039.48±162.47 | 1027.41±  156.18 | 1040.31±159.32 | 0.985 | 0.663 |
|  | R | 991.00±  128.66 | 930.25±  91.55 | 927.86±  141.16 | 0.016 | 0.719 |
| Caudal middle  frontal | L | 2230.81±301.85 | 2285.70±325.71 | 2169.24±337.18 | 0.242 | 0.271 |
|  | R | 2078.77±303.08 | 2122.41±363.42 | 1968.75±330.22 | 0.169 | 0.136 |
| Cuneus | L | 1460.37±186.55 | 1389.62±199.57 | 1410.69±254.23 | 0.306 | 0.509 |
|  | R | 1539.00±202.54 | 1475.00±203.71 | 1467.44±247.53 | 0.177 | 0.875 |
| Entorhinal | L | 407.33±  63.31 | 400.33±  61.20 | 365.24±  62.17 | 0.007 | 0.064 |
|  | R | 329.81±  65.57 | 332.87±  53.37 | 321.24±  60.52 | 0.612 | 0.617 |
| Fusiform | L | 3211.22±427.05 | 3167.25±307.37 | 3127.27±450.66 | 0.276 | 0.98 |
|  | R | 3158.59±418.40 | 3059.95±361.22 | 2911.58±423.57 | 0.003 | 0.316 |
| Inferior parietal | L | 4501.66±724.09 | 4506.45±507.77 | 4507.00±585.60 | 0.956 | 0.557 |
|  | R | 5314.00±825.97 | 5443.41±473.50 | 5410.27±619.99 | 0.512 | 0.754 |
| Inferior temporal | L | 3268.55±414.38 | 3255.83±254.43 | 3162.96±431.98 | 0.218 | 0.572 |
|  | R | 3087.07±489.98 | 3134.87±363.12 | 3022.31±424.24 | 0.373 | 0.56 |
| Isthmus cingulate | L | 996.70±  176.64 | 969.25±  155.74 | 977.51±  146.19 | 0.494 | 0.433 |
|  | R | 946.00± 130.08 | 932.75±  118.27 | 959.24±  119.82 | 0.949 | 0.122 |
| Lateral occipital | L | 4693.88±544.42 | 4426.83±605.26 | 4711.65±644.03 | 0.741 | 0.002 |
|  | R | 4538.33±559.17 | 4344.08±541.16 | 4550.34±475.95 | 0.927 | 0.006 |
| Lateral orbitofrontal | L | 2585.66±257.13 | 2594.62±281.83 | 2480.13±253.76 | 0.051 | 0.18 |
|  | R | 2550.85±205.77 | 2508.87±264.99 | 2414.82±252.98 | 0.008 | 0.258 |
| Lingual | L | 3026.81±423.37 | 2935.29±426.51 | 3064.69±501.61 | 0.86 | 0.126 |
|  | R | 3092.48±353.03 | 2963.00±430.04 | 3164.10±507.11 | 0.576 | 0.031 |
| Medial orbitofrontal | L | 1803.44±195.22 | 1849.41±263.53 | 1744.96±251.29 | 0.243 | 0.183 |
|  | R | 1771.44±162.47 | 1769.37±135.58 | 1704.20±238.44 | 0.151 | 0.305 |
| Middle temporal | L | 3064.70±368.49 | 3008.91±327.72 | 3072.24±336.51 | 0.917 | 0.201 |
|  | R | 3391.51±400.89 | 3307.29±278.72 | 3291.27±400.78 | 0.111 | 0.666 |
| Parahippocampal | L | 699.96±  110.90 | 703.33±  76.91 | 672.58±  115.03 | 0.161 | 0.448 |
|  | R | 656.03±  82.42 | 653.62±  69.77 | 648.93±  87.42 | 0.525 | 0.745 |
| Paracentral | L | 1251.88±141.20 | 1340.33±199.04 | 1281.93±147.59 | 0.634 | 0.293 |
|  | R | 1396.74±167.54 | 1497.95±215.59 | 1483.93±178.76 | 0.052 | 0.969 |
| Pericalcarine | L | 1365.11±201.20 | 1281.87±286.27 | 1355.58±298.56 | 0.883 | 0.174 |
|  | R | 1515.81±256.81 | 1473.00±259.60 | 1494.65±304.10 | 0.752 | 0.595 |
| Postcentral | L | 4071.88±415.41 | 4136.41±340.78 | 4031.69±412.90 | 0.554 | 0.545 |
|  | R | 3897.11±303.25 | 3941.95±346.69 | 3860.69±378.99 | 0.369 | 0.722 |
| Posterior cingulate | L | 1196.40±141.24 | 1190.54±139.25 | 1203.55±153.83 | 0.825 | 0.447 |
|  | R | 1197.37±176.98 | 1184.08±139.18 | 1250.82±184.01 | 0.163 | 0.043 |
| Precentral | L | 4705.88±434.99 | 4788.33±445.69 | 4623.82±457.09 | 0.244 | 0.314 |
|  | R | 4791.37±378.22 | 4888.83±511.12 | 4723.34±384.86 | 0.351 | 0.262 |
| Precuneus | L | 3672.07±312.14 | 3638.33±416.48 | 3645.93±410.50 | 0.495 | 0.451 |
|  | R | 3989.25±491.81 | 3858.37±558.77 | 3892.58±608.75 | 0.195 | 0.354 |
| Rostral middle frontal | L | 5424.55±632.93 | 5372.87±613.22 | 5360.82±715.08 | 0.427 | 0.602 |
|  | R | 5664.88±712.24 | 5630.37±561.55 | 5555.20±782.98 | 0.258 | 0.876 |
| Superior frontal | L | 6937.74±794.90 | 7021.33±765.38 | 6903.31±679.24 | 0.631 | 0.957 |
|  | R | 6733.29±659.55 | 6748.29±681.41 | 6680.37±620.70 | 0.462 | 0.793 |
| Superior parietal | L | 5268.00±604.44 | 5275.79±517.47 | 5178.06±652.27 | 0.343 | 0.87 |
|  | R | 5312.11±530.67 | 5260.58±565.42 | 5241.86±610.85 | 0.332 | 0.618 |
| Superior temporal | L | 3572.92±404.91 | 3687.16±359.21 | 3515.37±385.65 | 0.295 | 0.16 |
|  | R | 3429.92±324.91 | 3550.91±319.41 | 3283.62±294.97 | 0.015 | 0.001 |
| Supramarginal | L | 3829.63±486.30 | 3807.11±447.30 | 3721.24±485.66 | 0.259 | 0.335 |
|  | R | 3634.81±413.93 | 3561.62±440.82 | 3420.00±393.27 | 0.022 | 0.353 |
| Frontal pole | L | 202.25±  29.99 | 193.23±  28.88 | 202.44± 29.15 | 0.789 | 0.491 |
|  | R | 273.33± 43.48 | 276.70± 43.46 | 273.93± 42.50 | 0.984 | 0.867 |
| Temporal pole | L | 460.03± 52.07 | 449.54±59.69 | 432.82± 36.68 | 0.034 | 0.314 |
|  | R | 398.03± 51.13 | 398.45± 47.43 | 377.93± 47.56 | 0.106 | 0.186 |
| Transverse temporal | L | 427.29± 69.49 | 473.00± 76.31 | 415.93± 61.59 | 0.405 | 0.005 |
|  | R | 319.33± 56.96 | 337.29± 64.48 | 315.41± 41.46 | 0.753 | 0.222 |
| Insula | L | 2124.37±247.41 | 2101.16±169.22 | 2046.37±174.06 | 0.091 | 494 |
|  | R | 2151.51±290.49 | 2199.08±252.24 | 2133.10±196.87 | 0.829 | 0.496 |

**S3.** **Comparison of CT measurement of the other brain regions between BDI, FR-SB and HC**

*(BDI: bipolar disorder type I, FR-SB: siblings; HC: healthy controls, CT:cortical thickness)*

| ***Brain Regions (CT)*** | ***Hemisphere*** | ***BD***  ***(n=27)*** | ***FR-SB***  ***(n=17)*** | ***HC***  ***(n=29)*** | ***BD vs HC***  ***p values*** | ***FR-SB vs HC***  ***p values*** |
| --- | --- | --- | --- | --- | --- | --- |
| Bankssts | L | 2.47±0.15 | 2.49±0.18 | 2.47±0.19 | 0.688 | 0.522 |
|  | R | 2.56±0.16 | 2.54±0.23 | 2.50±0.21 | 0.193 | 0.338 |
| Caudal middle frontal | L | 2.52±0.24 | 2.49±0.20 | 2.48±0.17 | 0.921 | 0.607 |
|  | R | 2.42±0.14 | 2.48±0.18 | 2.47±0.19 | 0.481 | 0.669 |
| Cuneus | L | 1.73±0.14 | 1.73±0.11 | 1.73±0.16 | 0.893 | 0.901 |
|  | R | 1.70±0.12 | 1.78±0.12 | 1.75±0.20 | 0.332 | 0.393 |
| Entorhinal | L | 3.34±0.31 | 3.38±0.42 | 3.43±0.29 | 0.351 | 0.752 |
|  | R | 3.57±0.32 | 3.56±0.48 | 3.55±0.40 | 0.805 | 0.802 |
| Fusiform | L | 2.60±0.15 | 2.68±0.16 | 2.65±0.15 | 0.364 | 0.313 |
|  | R | 2.66±0.13 | 2.67±0.17 | 2.70±0.19 | 0.525 | 0.796 |
| Inferior parietal | L | 2.40±0.11 | 2.44±0.17 | 2.45±0.14 | 0.439 | 0.999 |
|  | R | 2.42±0.12 | 2.48±0.15 | 2.46±0.16 | 0.632 | 0.436 |
| Inferior temporal | L | 2.72±0.13 | 2.78±0.19 | 2.76±0.15 | 0.641 | 0.471 |
|  | R | 2.81±0.17 | 2.89±0.18 | 2.88±0.15 | 0.186 | 0.614 |
| Isthmus cingulate | L | 2.32±0.19 | 2.36±0.17 | 2.36±0.17 | 0.644 | 0.814 |
|  | R | 2.22±0.19 | 2.20±0.15 | 2.34±0.18 | 0.052 | 0.016 |
| Lateral occipital | L | 2.08±0.12 | 2.18±0.10 | 2.12±0.17 | 0.492 | 0.108 |
|  | R | 2.15±0.14 | 2.18±0.16 | 2.21±0.16 | 0.25 | 0.718 |
| Lateral  orbitofrontal | L | 2.53±0.17 | 2.60±0.24 | 2.58±0.16 | 0.72 | 0.485 |
|  | R | 2.50±0.16 | 2.54±0.14 | 2.52±0.14 | 0.939 | 0.557 |
| Lingual | L | 1.88±0.15 | 1.95±0.14 | 1.96±0.17 | 0.111 | 0.993 |
|  | R | 1.91±0.12 | 1.95±0.11 | 1.99±0.15 | 0.053 | 0.502 |
| Medial  orbitofrontal | L | 2.43±0.13 | 2.48±0.16 | 2.44±0.17 | 0.707 | 0.33 |
|  | R | 2.33±0.17 | 2.40±0.18 | 2.40±0.19 | 0.379 | 0.763 |
| Middle temporal | L | 2.82±0.16 | 2.86±0.14 | 2.85±0.13 | 0.681 | 0.761 |
|  | R | 2.84±0.12 | 2.88±0.19 | 2.90±0.18 | 0.23 | 0.807 |
| Parahippocampal | L | 2.69±0.30 | 2.71±0.27 | 2.72±0.23 | 0.754 | 0.988 |
|  | R | 2.66±0.23 | 2.74±0.32 | 2.74±0.21 | 0.26 | 0.942 |
| Paracentral | L | 2.25±0.13 | 2.34±0.19 | 2.30±0.17 | 0.391 | 0.411 |
|  | R | 2.29±0.15 | 2.35±0.20 | 2.32±0.16 | 0.791 | 0.381 |
| Pericalcarine | L | 1.46±0.09 | 1.49±0.10 | 1.54±0.31 | 0.18 | 0.428 |
|  | R | 1.48±0.11 | 1.51±0.09 | 1.56±0.27 | 0.104 | 0.418 |
| Postcentral | L | 2.00±0.11 | 2.02±0.13 | 2.00±0.14 | 0.751 | 0.377 |
|  | R | 1.97±0.11 | 1.97±0.12 | 1.99±0.13 | 0.901 | 0.938 |
| Posterior cingulate | L | 2.38±0.11 | 2.40±0.13 | 2.44±0.16 | 0.38 | 0.485 |
|  | R | 2.36±0.15 | 2.38±0.16 | 2.40±0.14 | 0.61 | 0.709 |
| Precentral | L | 2.45±0.11 | 2.47±0.18 | 2.47±0.18 | 0.919 | 0.959 |
|  | R | 2.43±0.11 | 2.44±0.18 | 2.46±0.18 | 0.823 | 0.763 |
| Precuneus | L | 2.25±0.14 | 2.28±0.14 | 2.28±0.14 | 0.649 | 0.993 |
|  | R | 2.24±0.14 | 2.29±0.14 | 2.28±0.14 | 0.454 | 0.721 |
| Rostral middle frontal | L | 2.28±0.14 | 2.37±0.15 | 2.37±0.15 | 0.048 | 0.789 |
|  | R | 2.24±0.12 | 2.27±0.10 | 2.33±0.15 | 0.049 | 0.261 |
| Superior frontal | L | 2.67±0.14 | 2.72±0.17 | 2.73±0.19 | 0.435 | 0.772 |
|  | R | 2.65±0.12 | 2.70±0.17 | 2.69±0.18 | 0.649 | 0.619 |
| Superior parietal | L | 2.11±0.12 | 2.16±0.13 | 2.14±0.17 | 0.675 | 0.575 |
|  | R | 2.10±0.11 | 2.13±0.14 | 2.13±0.14 | 0.707 | 0.679 |
| Superior temporal | L | 2.70±0.15 | 2.71±0.17 | 2.73±0.17 | 0.848 | 0.875 |
|  | R | 2.77±0.14 | 2.77±0.21 | 2.77±0.21 | 0.6 | 0.839 |
| Supramarginal | L | 2.48±0.12 | 2.53±0.17 | 2.51±0.16 | 0.9 | 0.359 |
|  | R | 2.45±0.11 | 2.50±0.16 | 2.51±0.15 | 0.4 | 0.905 |
| Frontal pole | L | 2.75±0.25 | 2.88±0.36 | 2.83±0.29 | 0.617 | 0.305 |
|  | R | 2.72±0.28 | 2.81±0.22 | 2.87±0.32 | 0.087 | 0.578 |
| Temporal pole | L | 3.52±0.26 | 3.63±0.34 | 3.64±0.32 | 0.265 | 0.935 |
|  | R | 3.73±0.30 | 3.72±0.44 | 3.77±0.37 | 0.839 | 0.752 |
| Transverse  temporal | L | 2.30±0.20 | 2.20±0.22 | 2.30±0.23 | 0.876 | 0.218 |
|  | R | 2.40±0.13 | 2.29±0.25 | 2.39±0.19 | 0.697 | 0.11 |
| Insula | L | 2.97±0.09 | 3.03±0.18 | 3.01±0.15 | 0.768 | 0.413 |
|  | R | 2.91±0.13 | 2.94±0.18 | 2.95±0.16 | 0.715 | 0.922 |

**S4. Comparison of SA measurement of the other brain regions between BDI, FR-SB and HC**

*(BDI: bipolar disorder type I, FR-SB:siblings, HC: healthy controls, SA:surface area)*

| ***Brain Regions***  ***(SA)*** | ***Hemisphere*** | ***BD***  ***(n=27)*** | ***FR-SB***  ***(n=17)*** | ***HC***  ***(n=29)*** | ***BD vs HC***  ***p values*** | ***FR-SB vs HC***  ***p values*** |
| --- | --- | --- | --- | --- | --- | --- |
| Bankssts | L | 1039.48±  162.47 | 1026.41±  135.38 | 1040.31±  159.32 | 0.931 | 0.675 |
|  | R | 991.00±  128.66 | 924.41±  83.50 | 927.86±  141.16 | 0.02 | 0.739 |
| Caudal middle frontal | L | 2230.81±  301.85 | 2271.05±  348.30 | 2169.24±  337.18 | 0.227 | 0.237 |
|  | R | 2078.77±  303.08 | 2206.11±3  86.96 | 1968.75±  330.22 | 0.117 | 0.016 |
| Cuneus | L | 1460.37±  186.55 | 1343.05±  200.34 | 1410.69±  254.23 | 0.335 | 0.193 |
|  | R | 1539.00±  202.54 | 1469.05±  209.81 | 1467.44±  247.53 | 0.201 | 0.832 |
| Entorhinal | L | 407.33±  63.31 | 381.88±  56.35 | 365.24±  62.17 | 0.006 | 0.627 |
|  | R | 329.81±  65.57 | 341.00±  55.73 | 321.24±  60.52 | 0.618 | 0.426 |
| Fusiform | L | 3211.22±  427.05 | 3080.41±  309.39 | 3127.27±  450.66 | 0.296 | 0.49 |
|  | R | 3158.59±  418.40 | 3072.11±  406.33 | 2911.58±  423.57 | 0.003 | 0.284 |
| Inferior parietal | L | 4501.66±  724.09 | 4620.88±  452.16 | 4507.00±  585.60 | 0.978 | 0.916 |
|  | R | 5314.00±  825.97 | 5491.23±  520.57 | 5410.27±  619.99 | 0.539 | 0.52 |
| Inferior temporal | L | 3268.55±  414.38 | 3241.47±  264.22 | 3162.96±  431.98 | 0.216 | 0.75 |
|  | R | 3087.07±  489.98 | 3090.70±  336.56 | 3022.31±  424.24 | 0.373 | 0.787 |
| Isthmus cingulate | L | 996.70±  176.64 | 971.58±  145.16 | 977.51±  146.19 | 0.528 | 0.404 |
|  | R | 946.00±  130.08 | 916.29±  96.89 | 959.24±  119.82 | 0.867 | 0.039 |
| Lateral  occipital | L | 4693.88±  544.42 | 4343.82±  651.35 | 4711.65±  644.03 | 0.692 | 0.001 |
|  | R | 4538.33±  559.17 | 4295.58±  596.18 | 4550.34±  475.95 | 0.882 | 0.002 |
| Lateral orbitofrontal | L | 2585.66±  257.13 | 2594.17±  298.84 | 2480.13±  253.76 | 0.057 | 0.196 |
|  | R | 2550.85±  205.77 | 2462.70±  260.51 | 2414.82±  252.98 | 0.01 | 0.681 |
| Lingual | L | 3026.81±  423.37 | 2816.29±  421.78 | 3064.69±  501.61 | 0.773 | 0.017 |
|  | R | 3092.48±  353.03 | 2821.52±  403.93 | 3164.10±  507.11 | 0.502 | 0.001 |
| Medial orbitofrontal | L | 1803.44±  195.22 | 1865.47±  291.87 | 1744.96±  251.29 | 0.261 | 0.142 |
|  | R | 1771.44±  162.47 | 1772.94±  127.01 | 1704.20±  238.44 | 0.163 | 0.381 |
| Middle temporal | L | 3064.70±  368.49 | 2963.64±  337.33 | 3072.24±  336.51 | 0.918 | 0.113 |
|  | R | 3391.51±  400.89 | 3290.17±  285.87 | 3291.27±  400.78 | 0.12 | 0.658 |
| Parahippocampal | L | 699.96±  110.90 | 694.76±  67.36 | 672.58±  115.03 | 0.18 | 0.538 |
|  | R | 656.03±  82.42 | 656.11±  74.27 | 648.93±  87.42 | 0.472 | 0.997 |
| Paracentral | L | 1251.88±  141.20 | 1343.17±  228.07 | 1281.93±  147.59 | 0.653 | 0.249 |
|  | R | 1396.74±  167.54 | 1522.58±  208.35 | 1483.93±  178.76 | 0.045 | 0.625 |
| Pericalcarine | L | 1365.11±  201.20 | 1215.64±  295.29 | 1355.58±  298.56 | 0.946 | 0.023 |
|  | R | 1515.81±  256.81 | 1407.41±  246.79 | 1494.65±  314.10 | 0.833 | 0.15 |
| Postcentral | L | 4071.88±  415.41 | 4133.05±  326.72 | 4031.69±  412.90 | 0.526 | 0.624 |
|  | R | 3897.11±  303.25 | 3925.00±  348.75 | 3860.69±  378.99 | 0.33 | 0.896 |
| Posterior cingulate | L | 1196.40±  141.24 | 1196.47±  145.54 | 1203.55±  153.83 | 0.796 | 0.497 |
|  | R | 1197.37±  176.98 | 1195.47±  142.76 | 1250.82±  184.01 | 0.197 | 0.083 |
| Precentral | L | 4705.88±  434.99 | 4802.05±  476.63 | 4623.82±  457.09 | 0.249 | 0.307 |
|  | R | 4791.37±  378.22 | 4991.88±  540.40 | 4723.34±  384.86 | 0.311 | 0.051 |
| Precuneus | L | 3672.07±  312.14 | 3580.47±  452.09 | 3645.93±  410.50 | 0.544 | 0.251 |
|  | R | 3989.25±  491.81 | 3793.58±  594.54 | 3892.58±  608.75 | 0.205 | 0.276 |
| Rostral middle frontal | L | 5424.55±  632.93 | 5396.05±  640.71 | 5360.82±  715.08 | 0.388 | 0.852 |
|  | R | 5664.88±  712.24 | 5597.17±  611.41 | 5555.20±  782.98 | 0.266 | 0.915 |
| Superior frontal | L | 6937.74±  794.90 | 7026.47±  847.46 | 6903.31±  679.24 | 0.573 | 0.938 |
|  | R | 6733.29±  659.55 | 6783.11±  774.67 | 6680.37±  620.70 | 0.435 | 0.923 |
| Superior parietal | L | 5268.00±  604.44 | 5197.70±  520.09 | 5178.06±  652.27 | 0.399 | 0.86 |
|  | R | 5312.11±  530.67 | 5149.41±  574.25 | 5241.86±  610.85 | 0.387 | 0.328 |
| ***Superior temporal*** | L | 3572.92±  404.91 | 3727.05±  386.76 | 3515.37±  385.65 | 0.248 | 0.083 |
|  | ***R*** | ***3429.92±***  ***324.91*** | ***3603.82±***  ***333.30*** | ***3283.62±***  ***294.97*** | 0.01 | ***<0.0001*** |
| Supramarginal | L | 3829.63±  486.30 | 3807.11±  447.30 | 3721.24±  485.66 | 0.292 | 0.918 |
|  | R | 3634.81±  413.93 | 3563.17±  449.50 | 3420.00±  393.27 | 0.028 | 0.43 |
| Frontal pole | L | 202.25±  29.99 | 193.23±  28.88 | 202.44±  29.15 | 0.885 | 0.17 |
|  | R | 273.33±  43.48 | 275.52±  43.41 | 273.93±  42.50 | 0.997 | 0.972 |
| Temporal pole | L | 460.03±  52.07 | 434.76±  58.92 | 432.82±  36.68 | 0.028 | 0.65 |
|  | R | 398.03±  51.13 | 396.29±  44.70 | 377.93±  47.56 | 0.106 | 0.299 |
| Transverse temporal | L | 427.29±  69.49 | 469.52±  84.37 | 415.93±  61.59 | 0.418 | 0.021 |
|  | R | 319.33±  56.96 | 346.05±  66.88 | 315.41±  41.46 | 0.716 | 0.104 |
| Insula | L | 2124.37±  247.41 | 2102.94±  185.72 | 2046.37±  174.06 | 0.098 | 0.54 |
|  | R | 2151.51±  290.49 | 2233.70±  277.61 | 2133.10±  196.87 | 0.812 | 0.325 |

**S5. The relationship between SA of LPT and clinical variables in BDI group**

*(LPT:left pars triangularis, SA:surface area, BDI:bipolar disorder type I)*

| ***Clinical variable*** | ***F value*** | ***p value*** |
| --- | --- | --- |
| Duration of illness | 2.29 | 0.15 |
| Total number of episodes | 0.001 | 0.97 |
| Episode frequency | 3.82 | 0.06 |
| Current lithium use | 3.07 | 0.09 |
| Current valproic acid use | 2.26 | 0.15 |
| Current atypical antipsychotic use | 0.63 | 0.44 |
